# Anæmic Vomiting

**Published:** 1907-02-16

**Authors:** 


### Points in Diagnosis.

### ANÆMIC VOMITING.

In many young women persistent vomiting after food, associated with pain and hyperæsthesia in the upper left quadrant of the abdomen, is of frequent occurrence. The age of the patients is from fifteen to twenty-five, the greater number of cases being about twenty. Sometimes the patient will tell one that she has kept nothing down for six weeks; and yet a striking feature of most of the cases is that there is no obvious emaciation. The patients now under consideration have had no profuse hæmatemesis, and yet the main difficulty in the diagnosis is to decide whether or not there is a gastric ulcer present. Many of the patients belong to the class of domestic servants, shop-assistants, or nursemaids. They are all of them more or less anæmic.

In cases of real gastric ulcer the abdominal pain is usually local to the epigastrium or to a spot in the left hypochondrium. In "anæmic vomiting" the pain is much more diffuse, and may indeed extend over almost the whole of the epigastrium and left side of the abdomen. The main point in differentiation between gastric ulcer and anæmic vomiting, however, is the effect of rest in bed upon the ability to retain food. Put the patient to bed, and begin giving full meat diet at once, or after twelve hours' rest in bed. If an ulcer be present, the full diet will continue to cause discomfort, or even acute pain. If the condition be "anæmic vomiting," full diet will be well borne after the patient has been resting for twelve hours. Should the patient get up again within a few days, and particularly if she should try to return to her work, the vomiting after food will come on again immediately, and will be almost as bad when milk only is taken as it would be on full diet. A return to bed will at once enable the patient to take full diet. This ability to take full diet when in bed, whilst vomiting recurs whenever the patient tries to work, suggests that the trouble is not really gastric at all, but rather cardiac. It seems that, owing to the poverty of the blood, the heart muscle readily dilates. With the patient at work, running up and down stairs, and carrying pails, etc., the heart is kept dilated; whereas in bed the temporary dilatation subsides at once. The close relationship between the stomach and the heart is well known, and is readily understood when one remembers that both heart and stomach are supplied by the same nerve, the vagus. Vomiting is often troublesome in cardiac valvular disease with failing compensation; flatulent dyspepsia readily produces cardiac symptoms in the form of palpitations. The relationship of heart to stomach in these cases of "anæmic vomiting" is not difficult to follow, and treatment must be directed towards the heart and the anæmia rather than the stomach. Rest in bed relieves the heart. Full diet to build up the strength of the patient is then possible, and iron in one form or another, combined with laxatives, will cure the anæmia, which is the primary trouble. The duration of the treatment in bed will vary with the degree of the anæmia, but it is seldom wise to allow the patient to be up and about in less than three weeks, or the anæmic vomiting will recur.

It is best not to give the iron in the form of pills, for so many of these remain undissolved and often appear in the motions. At first it is well to give one of the milder preparations. A very good form is

℞ Misturæ ferri compositæ (B.P.) ... ʒj. t.d.s.

and at the end of a week this may be changed for

℞ Liquoris ferri perchloridi ... ... ♏xv.

Glycerini ... ... ... ʒj.

Infusi quassiæ ad ... ... ... ʒj. t.d.s.

If the constipation is very severe, magnesium sulphate may be added to one or more of the doses each day; or another good prescription is—

℞ Ferri sulphatis ... ... ... gr. iv.

Magnesii sulphatis ... ... ... gr. xl.

Acidi sulphurici diluti ... ... ♏x.

Aquæ chloroformi ad ... ... ʒj. t.d.s.

In many cases iron in the above forms may cause indigestion, in which case a very suitable form is

℞ Alginoid iron ... ... ... ... gr. x.

given as *powder*, in cachet, four times a day. It is not soluble in acids, but is dissolved in alkaline media. It therefore remains as powder until it has passed through the pylorus and has reached the pancreatic secretion in the duodenum.

It is almost unnecessary to mention the necessity of using a tooth-brush after each dose of an iron mixture to prevent discolouration of the teeth. The blackening of the teeth by iron is very well known, but it may be quite avoided by the use of a tooth-brush after each dose of the medicine.

